# The Politics of Vaccination: A Global History

**DOI:** 10.3201/eid2411.181045

**Published:** 2018-11

**Authors:** Laura E. Power

**Affiliations:** University of Michigan, Ann Arbor, Michigan, USA

**Keywords:** Alexander Langmuir, bacteria, book review, history, politics, public health, respiratory infections, vaccination, viruses, William Foege

Nothing happens in a vacuum, including public health interventions, which do have unintended consequences; whether those consequences are positive or negative may depend on the eye of the beholder. The Politics of Vaccination: A Global History, edited by Christine Holmberg, Stuart Blume, and Paul Greenough (Figure), describes the political and cultural contexts of vaccination efforts throughout history. The book examines the tensions existing between national independence and global interdependence, top-down program implementation and grassroots development, and individual rights and the common good. Each chapter tells the story of a specific vaccination program, and although time and place differ, when taken together, they highlight the push-and-pull that exists between individuals, nations, and multinational agencies in this context. 

**Figure:**
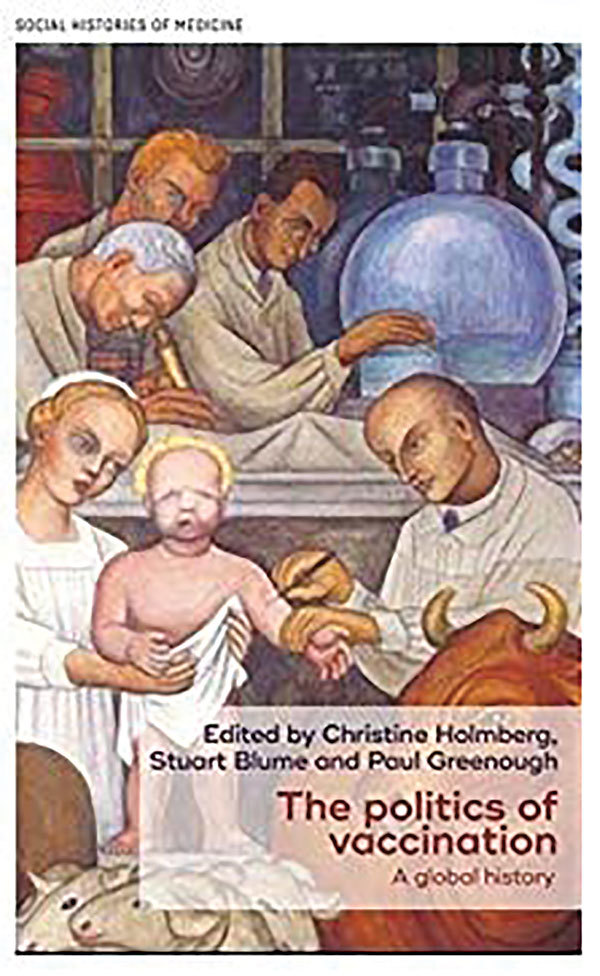
The Politics of Vaccination: A Global History

The book is divided into three sections. The first demonstrates how national identity is shaped by the way the nation’s people respond to vaccine campaigns and interventions, especially when initiated by an outside agency. The second section traces the rise and fall over time of national vaccine production and the eventual transition to and reliance on multinational agencies to meet national needs, other than in outlier countries like Brazil and Japan. These accounts leave the reader unsure of how reliable these agencies will remain for individual countries in the future. The third section studies the pitfalls of the top-down approach in surveillance and prevention campaigns and underlines the difficulty of balancing individual rights with obligations to protect the public. 

These three sections are outlined in an introduction by the editors, which lays out a map for the reader to follow throughout the book. The commentary in the afterword, by asserting that the top-down approach of vaccine campaigns initiated by outside global leaders in developing nations results in significant collateral damage and ignores the national priorities, finalizes the tone for the book as a whole. 

Readers, regardless of their stances on the issues raised, will be challenged to think about the different lenses through which individuals or nations might see vaccine campaigns, and even public health interventions in general. Medical historians and anthropologists will appreciate the book’s perspectives on past experiences, which will be thought-provoking to public health practitioners and global health activists as well. Readers will enjoy the rich detail and historical references in each chapter and be intrigued by the character studies of textbook global health heroes, such as Dr. Alexander Langmuir and Dr. William Foege. The expert information can help public health practitioners consider in advance the effects, including potential unintended consequences, that large-scale interventions may have on a population. And, although the book may challenge those looking for a more straightforward account of the history of vaccination, most readers will find topics of interest within the various time periods and countries described. The Politics of Vaccination: A Global History, through its fresh and sometimes provocative perspective on global public health, reminds readers of the importance of political and cultural context when practicing disease surveillance and prevention. 

